# Epididymoorchitis as the First Finding in Patients with Brucellosis

**DOI:** 10.1155/2013/765023

**Published:** 2013-12-26

**Authors:** Ayhan Karaköse, Mehmet Bilgehan Yuksel, Özgü Aydoğdu, Aziz Ahmad Hamidi

**Affiliations:** ^1^Izmir University School of Medicine, Department of Urology, 35200 Izmir, Turkey; ^2^Celal Bayar University, School of Medicine, Department of Urology, 45040 Manisa, Turkey; ^3^Mus State Hospital, Department of Infectious Diseases, 49100 Mus, Turkey

## Abstract

*Purpose*. Acute scrotal pain as the first symptom of brucellosis is rarely observed. We aimed to evaluate the data of male patients with brucellosis and epididymoorchitis as the initial diagnosis. *Material and Methods*. The data of seven patients presented with testicular pain, hyperemia, swelling, and increased fever were reviewed. Concomitant focal diseases as well as clinical, laboratory, and radiological findings were retrospectively evaluated. *Results*. The mean age of the patients was 22.28 ± 7.78 (16–35) years. All patients presented with scrotal pain, swelling, and increased sweating. Additional findings included fever, asthenia, arthralgia, dysuria, shiver and rash, weight loss, and vomiting in 6, 5, 4, 4, 3, 2, and 1 patient, respectively. In all of 7 patients, the agglutination tests of Rose-Bengal and Wright were positive. Coombs test was positive only in 3 patients. The patients underwent antibiotic and conservative treatment. No relapse was observed following the treatment. *Conclusion*. In endemic regions, epididymoorchitis caused by brucellosis should be considered in the differential diagnosis of patients presenting with acute scrotal pain. Clinical and serological findings are sufficient for the diagnosis. Conservative management combined with antibiotic therapy is adequate for managing brucellar epididymoorchitis.

## 1. Introduction

Brucellosis, which is also called Mediterranean or Malta fever, is an endemic enzootic disease and can involve various organ systems. Brucellosis constitutes a major health and economic problem in many parts of the world, encompassing the Mediterranean countries and Middle East [1, 2]. Epididymoorchitis is a focal form of human brucellosis with an incidence of 2–20% in patients with brucellosis [3, 4]. Brucella species cause granulomatous orchitis usually presenting as an acute or chronic unilateral swelling of the testis. Epididymoorchitis can be seen as a subsequent part of systemic disease after the previous diagnosis of Brucellosis. However, although the occurrence of Brucellar epididymoorchitis (BEO) as the presenting finding is an extremely rare manifestation of Brucellosis, patients can rarely apply to the clinicians with acute scrotum as an initial finding. Thus, BEO must be considered in the differential diagnosis of acute scrotum in endemic regions [5–9]. In the recent study, we aimed to describe the data of 7 patients who had no previous diagnosis of Brucellosis and were with BEO as a single, primary manifestation of the disease in endemic region of Mus, Turkey, and to present the importance of considering the BEO in the cases of acute scrotum in endemic regions of Brucellosis.

## 2. Materials and Methods

The data of 7 patients who had no previous diagnosis of Brucellosis and applied to our out-patient clinic with only testicular pain, hyperemia, swelling, and increased fever as an acute scrotum case between February 2011 and February 2012 were reviewed. Haemogram, C-reactive protein (CRP), erythrocyte sedimentation rate (ESR), urine analysis, and scrotal power duplex ultrasound scan (US) findings were retrospectively evaluated. Initially, all patients were diagnosed as nonspecific orchitis and treated with antibiotics and analgesic. In the following period, the patients consulted the infectious disease clinic since the complaints were not improved. All patients were hospitalized and followed in the infectious disease clinic to investigate the potential etiologic factors of antibiotic resistant nonspecific epididymoorchitis. Haemogram, urine culture, CRP, ESR, ALT, blood culture, Rose-Bengal test, Wright agglutination test, and Coombs test were performed in all patients. In addition, scrotal color Doppler US and MRI were performed for the differential diagnosis of testicular masses.

## 3. Results

The 7 patients had no previous diagnosis of brucellosis when they applied to our outpatient clinic. The mean age of the patients with a diagnosis of BEO was 22.28 ± 7.78 (16–35) years. Totally 28 male patients have been hospitalized for brucellosis in infectious disease clinic between February 2011 and February 2012 (mean age; 34.64 ± 15.11 (16–70) years). BEO rate in all brucellosis cases was calculated as 25%. All BEO cases presented with acute scrotal pain, swelling, and increased sweating. Additional findings included fever, asthenia, arthralgia, dysuria, shiver and rash, weight loss, and vomiting (Table 1).

Coexisting focal disease included osteoarticular involvement, spondylitis, sacroiliitis, peripheral arthritis, and hepatitis in 4, 3, 2, 2, and 1 patients, respectively (Table 2).

Laboratory investigations showed that all of BEO patients had positive Rose-Bengal and Wright agglutination tests. Coombs test was positive in 3 of 7 patients. Other abnormal laboratory findings included CRP > 5 mg/dL (6 patients), ESR > 20 mm/h (5 patients), WBC > 10.500 WBCs/mm^3^ (3 patients), ALT > 40 IU/L (3 patients), ALP > 150 IU/L (1 patient), and PLT < 150.000 Platelets/mm^3^ (1 patient) (Table 3). Blood and urine cultures were clear in all patients.

**Figure 1:**
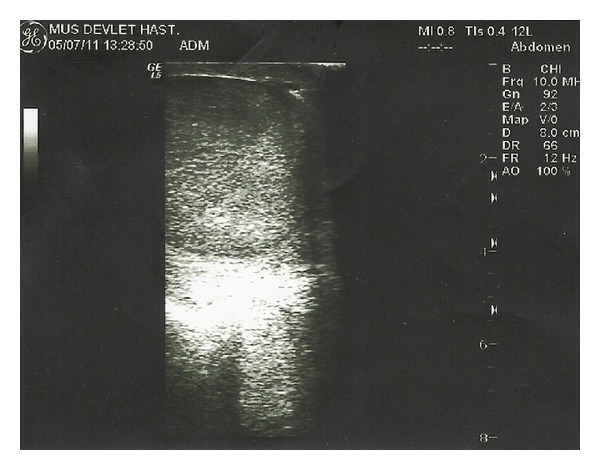
The scrotal color Doppler US image of a patient with BEO.

**Figure 2:**
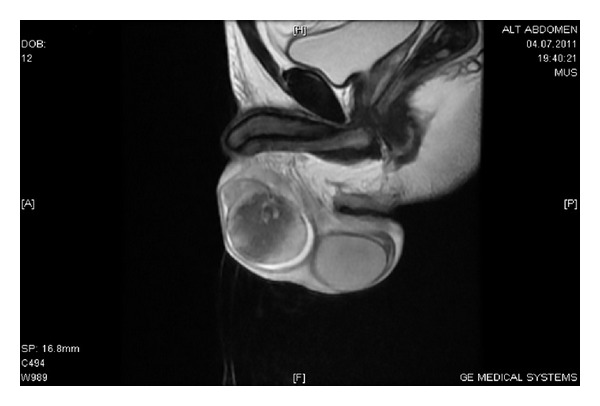
The scrotal MRI image of a patient with BEO.

The scrotal color Doppler US and scrotal MRI scan were performed for the differential diagnosis of possible testicular abscess and masses (Figures 1 and 2). While two patients were treated with doxycycline 2 × 100 mg PO and rifampicin 1 × 600 mg IV, 5 patients underwent streptomycin 1 × 1 gr IM and doxycycline 2 × 100 mg PO treatment over 6 weeks. 3 BEO patients with coexisting spondylitis had also sacroiliitis at the same time. These patients were treated with streptomycin 1 × 1 gr IM and doxycycline 2 × 100 mg PO combination during 2 weeks and subsequently doxycycline 2 × 100 mg and rifampicin 1 × 600 mg treatment regime was used in the following 3 months. Three patients with no skeletal involvement were treated with streptomycin 1 × 1 gr IM during 2 weeks and doxycycline 2 × 100 mg PO during 6 weeks.

While the mean hospitalization time of patients with the diagnosis of BEO and brucellosis was 10 ± 7.61 (4–22) days, it was 9.67 ± 6.03 (1–22) days in 28 patients who were diagnosed with brucellosis.

## 4. Discussion

Brucellosis which is caused by *Brucella* spp. and involves various organ systems, is an endemic enzootic disease. It is presented with many findings which may potentially be observed in several other diseases [1, 2]. The most frequent (2–20%) genitourinary complication of brucellosis is epididymoorchitis [3, 4]. While BEO can occur as a separate disease with no symptoms of systemic disease, it can be seen in the relapses of cases that were inadequately treated. Unilateral involvement is commonly seen in BEO. Urine analysis and culture are generally sterile [8, 10]. Yetkin et al. evaluated 186 brucellosis patients during 4 years and diagnosed 17 BEO in 186 [11]. The authors reported that 88% of BEO cases had unilateral involvement. BEO mimics testicular malignancy and/or tuberculosis with granulomatous inflammation in testis. Brucellosis, tuberculosis, infections, and trauma should be considered in the differential diagnosis of granulomatous orchitis [12, 13]. Prostate secretion and sperm cultures can be used in the diagnosis of BEO. In the previous literature, the proliferation in sperm culture of a BEO case was reported [14]. Acute scrotal pain, swelling and erythema, and unilateral involvement are characteristic findings of BEO patients reported in the previous papers [12, 15]. In the recent study, unilateral acute scrotal pain and swelling were observed in all patients.

Although the patients with brucellosis generally present with nonspecific symptoms, specific findings related to the involved organ system may also be observed. The previous literature showed that the most common complaints related to brucellosis are fever (61.2–93%), asthenia (76–97.5%), increased sweating (70.9–91%), and arthralgia (57–65%). Hepatomegaly (8.6–34.5%), splenomegaly (10.7–25.5%), lymphadenomegaly (7–11.4%), and arthritis (5.7–40%) may potentially be identified [10, 13, 15–17]. Physical examination shows fever, scrotal pain/swelling, and hepatosplenomegaly in 74–100%, 91–100%, and 25–31% of patients with BEO, respectively. Changes in serum CRP, ESR, ALT, AST, leucocyte (leukopenia/leukocytosis), Hb (anemia), and platelet (thrombocytopenia) may be seen in patients with BEO [15, 18–20]. In our study, all BEO patients had scrotal pain, swelling, and increased sweating. Fever, asthenia, arthralgia, dysuria, shiver, rash, weight loss, and vomiting were observed in 6, 5, 4, 4, 3, 3, 2, and 1 patients, respectively. In addition osteoarticular involvement, spondylitis, sacroiliitis, and hepatitis were determined in 4, 3, 2, and 1 patients, respectively.

In the suspicion of BEO, Brucellar agglutination tests and scrotal color Doppler US are crucial diagnostic methods for the differential diagnosis of epididymoorchitis in endemic regions of brucellosis [21]. In a previous study, 84 patients with epididymoorchitis were evaluated and BEO was determined in 14 of 84 cases (16.6%). Although brucellosis agglutination test was positive in all cases, the proliferation in blood culture was found only in 4 (28.5%) patients [7]. The proliferation rate in blood culture has been previously reported between 14% and 69% in the literature [12, 15, 20, 22]. The recent study revealed that Rose-Bengal and Wright agglutination tests were positive in all cases. While Coombs test was positive in 3 patients, no proliferation was present in blood and urine cultures.

## 5. Conclusion

Clinicians should be alert for BEO in the differential diagnosis of nonspecific epididymoorchitis, especially in endemic regions for brucellosis. Clinical and serological data are sufficient for the diagnosis of BEO. Conservative management combined with antibiotic therapy is adequate for the treatment.

## Figures and Tables

**Table 1:** Clinical findings of patients diagnosed with BEO.

Finding	*n*	%
Scrotal pain and swelling	7	100
Fever (temperature, ≥38°C)	6	85.71
Sweating	7	100
Asthenia	5	71.42
Arthralgia	4	57.14
Shiver	3	42.85
Dysuria	4	57.14
Weight loss	2	28.57
Rash	3	42.85
Vomiting	1	14.28

**Table 2:** Coexisting focal disease in patients diagnosed with BEO.

Focal disease	*n*	%
Osteoarticular involvement	4	57.14
Sacroiliitis	2	28.57
Hepatitis	1	14.28
Spondylitis	3	42.85
Peripheral arthritis	2	28.57

**Table 3:** Abnormal laboratory findings.

	*n*	%
Positive Rose-Bengal test	7	100
Positive Wright agglutination (≥1 : 160)	7	100
Positive Coombs test	3	42.85
CRP > 5 mg/dL (mean CRP 14.99 ± 10.70 (2.17–32.50) mg/dL)	6	85.71
ESR > 20 mm/h (mean ESR 24.57 ± 21.76 (2–58) mm/h)	5	71.42
WBCs/mm^3^ > 10.500	3	42.85
Platelets/mm^3^ < 150.000	1	14.28
ALP > 150 IU/L	1	14.28
ALT > 40 IU/L	3	42.85
